# MACC1 driven alterations in cellular biomechanics facilitate cell motility in glioblastoma

**DOI:** 10.1186/s12964-020-00566-1

**Published:** 2020-06-05

**Authors:** Tim Hohmann, Urszula Hohmann, Marc R. Kolbe, Mathias Dahlmann, Dennis Kobelt, Ulrike Stein, Faramarz Dehghani

**Affiliations:** 1 Institute of Anatomy and Cell Biology, Martin Luther University Halle-Wittenberg, Grosse Steinstrasse 52, 06108 Halle, Saale Germany; 2 Experimental and Clinical Research Center, Charité Universitätsmedizin Berlin and Max-Delbrück-Center for Molecular Medicine in the Helmholtz Association, Robert-Rössle-Straße 10, 13125 Berlin, Germany; 3 German Cancer Consortium (DKTK), Im Neuenheimer Feld 280, 69120 Heidelberg, Germany

**Keywords:** MACC1, Glioblastoma, Adhesion, Elasticity, Migration, Biomechanics

## Abstract

**Background:**

Metastasis-associated in colon cancer 1 (*MACC1*) is an established marker for metastasis and tumor cell migration in a multitude of tumor entities, including glioblastoma (GBM). Nevertheless, the mechanism underlying the increased migratory capacity in GBM is not comprehensively explored.

**Methods:**

We performed live cell and atomic force microscopy measurements to assess cell migration and mechanical properties of *MACC1* overexpressing GBM cells. We quantified *MACC1* dependent dynamics of 3D aggregate formation. For mechanistic studies we measured the expression of key adhesion molecules using qRT-PCR, and MACC1 dependent changes in short term adhesion to fibronectin and laminin. We then determined changes in sub-cellular distribution of integrins and actin in dependence of *MACC1*, but also in microtubule and intermediate filament organization.

**Results:**

*MACC1* increased the migratory speed and elastic modulus of GBM cells, but decreased cell-cell adhesion and inhibited the formation of 3D aggregates. These effects were not associated with altered mRNA expression of several key adhesion molecules or altered short-term affinity to laminin and fibronectin. *MACC1* did neither change the organization of the microtubule nor intermediate filament cytoskeleton, but resulted in increased amounts of protrusive actin on laminin.

**Conclusion:**

*MACC1* overexpression increases elastic modulus and migration and reduces adhesion of GBM cells thereby impeding 3D aggregate formation. The underlying molecular mechanism is independent on the organization of microtubules, intermediate filaments and several key adhesion molecules, but depends on adhesion to laminin. Thus, targeting re-organization of the cytoskeleton and cell motility via MACC1 may offer a treatment option to impede GBM spreading.

Video Abstract

## Background

Tumors are one of the most common causes of death worldwide with rising numbers [1, 2]. One highly lethal tumor entity is glioblastoma (GBM), with a median survival time of approximately 14 month [3]. The poor prognosis for GBM patients is caused by its resistance to standard therapy. Its high heterogeneity and the diffuse infiltration pattern into the adjacent brain tissue renders current therapy insufficient [4–6]. Especially the migratory capabilities of GBM cells pose a major obstacle in therapy, as cells distant from the main tumor mass escape resection and will form new tumors. The biological processes regulating cell migration are governed by the cytoskeleton and controlled by e.g. the c-Met/hepatocyte growth factor (HGF) axis [7–9]. Metastasis-associated in colon cancer 1 (*MACC1*) has been shown to be important for signaling through c-Met [10–13].

*MACC1* was first identified in 2009 as a prognostic biomarker for metastasis formation in colorectal cancer [12]. It correlates with a multitude of pro-tumoral functions, ranging from increased migration and proliferation to an association with drug-resistance [13]. Confirming the results in colorectal cancer, MACC1 expression is associated with a worse prognosis in various solid tumor types, including GBM [12, 13]. Furthermore, *MACC1* expression is increased in glioma, when compared to healthy brain tissue [14, 15]. *MACC1* correlates with the staging of gliomas and is associated with their potential to form recurrences [16]. *MACC1* induces a more aggressive behavior of glioma and GBM cells by increasing proliferation and migration and decreasing apoptosis [14, 16–19]. While these cellular effects of *MACC1* in glioma seem well established bio-mechanical studies were not yet performed.

The current study aims to characterize the effect of *MACC1* in glioblastoma on three hierarchical levels: multicellular, cellular and subcellular. On the multicellular level we determined the adhesion dynamics of GBM cell lines via the formation process of 3D aggregates. On the cellular level we measured motility, adhesion to specific extra-cellular matrix components and mechanical properties of single cells. Regarding the subcellular level we evaluated the organization of the cytoskeleton and the amount and distribution of specific adhesion molecules inside of the cell.

## Materials and methods

### Cell culture

U251 cells were purchased from the American Type Culture Collection (Manassas, VA, USA) and U138 cells were obtained from Cell Lines Service (Cell Lines Service, 300,363, Eppelheim, Germany). Cell lines were authenticated using Multiplex Cell Authentication by Multiplexion (Heidelberg, Germany) as described previously [20]. The single nucleotide polymorphism profiles matched known profiles or were unique. The generation of MACC1 overexpressing cell lines U138/MACC1 and U251/MACC1 and their respective controls (U138/EV and U251/EV) was previously described [16]. For experiments inhibiting the transcriptional target of MACC1, c-MET, we added 500 nM of crizotinib (Active Biochem, Hong Kong, China, A-1031) 24 h prior to the start of the experiments.

All cell lines were cultured in 87% (v/v) low glucose DMEM (1 g/l glucose; Gibco, 31,885–023), supplemented with 10% (v/v) FBS (Gibco, 10,500–064), 2% (v/v) non-essential amino acids (Biochrome, K0293) and 1% (v/v). penicillin/streptomycin (Gibco, 15,140–122).

### Single cell motility

For time lapse microscopy 1000 cells were seeded in a 6-well plate 24 h prior to the start of experiments. Images were taken with a microscope (Leica DMi8, Leica, Wetzlar, Germany) equipped with temperature (37 °C) and CO_2_ regulation (5% (v/v)). The experiments were conducted as described previously [21, 22]. Thereby we determined the parameters contact area to the substrate and mean speed.

### Fibronectin and laminin coating

For coating with fibronectin (FN), concentrated sulfuric acid was applied for 2 h onto the coverslips. Afterwards the acid was removed, and the coverslips rinsed three times with PBS. 300 μl of 10 μg/ml FN (Merck Millipore, Burlington, MA, USA 341635) solution was added for 24 h [23, 24], before coverslips were rinsed three times with PBS. For laminin (LN) coating LN (Sigma Aldrich, St. Louis, MO, USA, L4544) was used according to manufacturer instructions. Briefly, LN was diluted to a 10 μg/ml solution in HBSS (Thermo Fisher, Carlsbad, CA, USA) and added onto the coverslips for 2 h followed by washing with PBS.

Cantilevers for atomic force microscopy experiments were directly coated with 10 μg/ml FN or LN for 24 h. Successful coating was verified by immunocytochemistry with FN or LN antibodies.

### Atomic force microscopy

For measuring mechanical properties of glioma cells the Young’s modulus was determined, using an atomic force microscope (AFM; Bruker, Billerica, MA, USA, Bioscope Catalyst), as described previously [21]. Cells were allowed to adhere to a petri dish for 15 min before the start of the experiment. Single cells were measured with a tip-less cantilever (Arrow-TL2, Nanoworld, Neuchatel, Switzerland) using a force of 3 nN for determining the Young’s modulus with the Hertz model. For measuring adhesion laminin or fibronectin coated cantilevers were used and cells were indented with a force of 0.5 nN with a contact time of 30 s before cantilever retraction. For measuring adhesion the number of discrete rupture events was counted.

### 3D tumor aggregate formation assay

For cultivation of 3D tumor aggregates the liquid-overlay method was used. Therefore 50,000 cells were plated in 96-wells coated with 4% (w/v) agarose and allowed to aggregate for 6 h before starting the imaging process. The delay was necessary to determine the final position of an emerging aggregate. Imaging was performed for 72 h, and images were taken every 15 min. Image analysis was performed with self-developed software and is described below. As read-outs we determined the aggregate size, its brightness relative to the background, its circularity and the brightness as a function of its distance to the aggregate center (Additional file 1). Together with the AFM measurements the aggregation assay allows the estimation of the cell-cell adhesion energy (supplementary materials) [25].

### Image analysis of 3D tumor aggregate formation assays

For image analysis we used a custom written MatLab (The MathWorks, Natick, USA) script determining the edge of the 3D aggregate using the Chan-Vese image segmentation model [26], tracking each 3D aggregate over time. For each 3D aggregate we analyzed its size, defined as its pixel count, its brightness relative to the background, as a measure for the compactness of the 3D aggregate and its shape. For assessing the shape we calculated the circularity of the 3D aggregate as the ratio between the area of a perfect circle with a circumference that is equal to the outline of the 3D aggregate and the 3D aggregate area. For a 3D aggregate with area *A* and circumference *U* the circularity *c* is given as: c = 4*π*A/U^*2*^.

Despite this global approach we performed a local analysis of the 3D aggregate density relative to its center. Therefore, we calculated the distance of each pixel inside the 3D aggregate to its center and calculated the mean intensity for each distance value and normalized the result to the background intensity (Additional file 1 A and B). Afterwards, local minima were identified in the intensity over distance plot (Additional file 1 C) using a Gaussian fit and its proportion to the 3D aggregate, weighted with the intensity values, was determined. This value gives an estimate of the proportion of three dimensional structures relative to the whole 3D aggregate.

### Estimation of cell-cell adhesion energies from 3D tumor aggregate formation assays

For estimation of the cell-cell adhesion using the aggregation assay, we used a modified model introduced by Frasca et al. [25]. They concluded from their measurements that the adhesion energy *W* gained when establishing cell-cell contact can be derived by the following formula:

$$ W=\frac{2 Ed}{\sqrt{3}\pi \left(1-{\nu}^2\right)}{\left(1-{\left(\frac{p_0}{p_{\infty }}\right)}^{1/3}\right)}^{3/2} $$

Here *E* is the Young’s modulus of a single cell, *d* the initial cell-cell distance, *ν* the Poisson ratio, *p*_*0*_ and *p*_*∞*_ the initial and equilibrium compacity. The compacity is a geometric parameter describing the 3D aggregate and given as:

$$ p=N\ast \frac{V_{Cell}}{V_{Sphaeroid}} $$

With *N* as the number of cells in the aggregate and *V* as the volume of the cell or 3D aggregate, respectively.

The Young’s modulus *E* has been measured using the AFM, together with the average diameter of the cell, giving a good estimate of the initial cell-cell distance *d*. This assumption is valid as the diameter of the cells was measured for AFM experiments 15 min after seeding. Additionally, the number of initially seeded cells *N* is known. The 3D aggregate volume can be calculated using V = A*h, with the 3D aggregate cross-sectional area *A* and its average height *h*. The area *A* was directly measured, while the height *h* can only be indirectly assessed, as follows: *h ~ 1-I*, with the measured relative intensity *I*.

Consequently, the energy of adhesion can be assessed as follows:

$$ W=\frac{2 Ed}{\sqrt{3}\pi \left(1-{\nu}^2\right)}{\left(1-{\left(\frac{A_{\infty }\ \left(1-{I}_{\infty}\right)}{A_0\ \left(1-{I}_0\right)}\right)}^{1/3}\right)}^{3/2} $$

This equation assumes that N_0_*V_cell 0_ ≈ N_∞_*V_cell ∞_. The values used for calculation of adhesion energies can be found in the supplement.

### Immunofluorescence and immuncytochemical staining

For assessing βIII-tubulin, GFAP (glial fibrillary acidic protein) and vimentin organization, 50,000 cells were seeded on glass coverslips, cultured for 24 h, fixed with 4% paraformaldehyde for 10 min and labelled by immuncytochemistry. For immunocytochemistry cells were fixed and treated with methanol/H_2_O_2_ (100:1) for 30 min, than washed thrice with 0.02 M PBS for 10 min before blocking unspecific bindings using normal goat serum (diluted 1:20 in 0.02 M PBS/0.3% (v/v) Triton). Afterwards, the antibody for GFAP (BD Biosciences, Franklin Lakes, NJ, USA, 556330, 1:200), vimentin (Cell Signaling, Danvers, MA, USA, EPR3776, 1:100) or βIII-tubulin (Abcam, Cambridge, UK, ab18207, 1:1000) was added for 16 h, respectively. Samples were washed thrice with PBS, incubated with ExtrAvidin-peroxidase (Sigma Aldrich, St. Louis, MO, USA, E2886, 1:100) and washed twice with PBS before 5 min incubation with DAB. Afterwards, samples were covered with entallan.

For analysis of actin structure and integrin distribution, cells were labeled with phalloidin (actin), antibodies against integrin β1 and α5 and DAPI to stain nuclei. 50,000 cells were placed on uncoated, fibronectin or laminin coated glass coverslips and incubated for 30 min or 24 h till the fixation with 4% paraformaldehyde for 10 min. Afterwards cells were fluorescently labelled. For integrin labelling unspecific bindings was blocked with normal goat serum for 30 min before anti-integrin β1 (Merck Millipore, MAB2252, 1:500) antibody was applied for 16 h. Afterwards, the coverslips were washed three times with PBS and incubated for 12 h with anti-integrin α5 antibody (Abcam, Cambridge, UK, ab150361, 1:250). After washing with PBS the first secondary antibody goat anti-mouse AlexaFluor® 568 conjugated (Thermo Fisher, A-11031) was applied for 1 h before washing and incubation with second secondary antibody: goat anti-rabbit AlexaFluor® 633 conjugated (Thermo Fisher, A-21071). For actin labelling a phalloidin-488 staining was used. Cells were washed twice for 10 min in PBS, than incubated with 0.1% PBS/Triton for 5 min and blocked with 1% bovine serum albumin. Phalloidin-488 252 (2.5 μl/100 μl BSA solution, Thermo Fisher Scientific, 253 Waltham, MA, USA, A12379) was applied for 20 min. For the visualisation of nuclei 4′,6-Diamin-2-phenylindol (DAPI, 1:10000, Sigma Aldrich, D9542) was used. The stained cells were washed with PBS and distilled water and covered with DAKO mounting medium (DAKO, Santa Clara, CA, USA).

Fluorescence images were acquired with a 63× objective using a confocal laser scanning microscope (LSM 710 Meta, Zeiss, Göttingen, Germany). For detection of DAPI, phalloidin, Alexa 568 and Alexa 633 the following excitation wavelengths were used: 405 nm, 488 nm, 543 nm and 639 nm, respectively. Emission was detected in the range of Δλ = 400–500 nm (DAPI), Δλ = 510–550 nm (Phalloidin), Δλ = 585–615 nm (Alexa 568) and λ > 660 nm (Alexa 633).

### Image analysis of integrin distribution

For evaluation of the integrin distribution inside the cells we used a custom written MatLab (The MathWorks, Natick, USA) script. To detect the outlines of single cells in the CLSM images, three different approaches were used to generate binary images using the image channel containing the actin staining. We used the sobel operator on the median filtered image, with subsequent morphological operations (dilatation, erosion, removal of small objects) to obtain a binary image. Additionally, the Chan-Vese model was used for segmentation [26], with subsequent morphological operations (dilatation, erosion, removal of small objects). The last approach used was k-means segmentation. Therefore, the actin channel was filtered thrice with a Gaussian filter and k-means clustering to identify pixels of 4 classes was performed: bright, medium bright, dim, background pixels [27]. Bright, medium bright and dim pixels were combined with a logical OR to the segmented image. Combining the three obtained binary images with a logical OR resulted in the final binary image used for edge determination. The combination of these three approaches led to significantly more robust results than each single approach.

To calculate the distribution of integrins inside the cell the distance of each pixel inside the cell to the closest cell boundary pixel was calculated. Afterwards, the mean intensity of pixels for each distance rounded to the closest integer was calculated and finally all values were normalized to the area under the curve. This allows the comparison of integrin distributions inside of cells independent of labeling intensity and cell shape.

### Image analysis of actin structures

For evaluation of actin cytoskeletal alterations we used an image coherency based approach as described elsewhere [28]. This approach assumes that the overall structure can be understood as the sum over all local structures of actin fibers inside the cell. Thereby, the structure density can be obtained as the structuredness normalized to the cell area. The images were analyzed using a self-written MatLab (The MathWorks, Natick, USA) script, as published before [29].

To additionally evaluate possible changes in the distribution of actin structures we used a machine learning approach based on support vector machines (SVM) with RBF kernel to identify four different types of actin structures: cortical fibers, stress fibers, protrusive actin and punctuate actin [27]. Therefore, nine images of each U138 EV and U251 EV cells were classified manually, containing 3 images of cells plated on glass, fibronectin or laminin, respectively. This classification was used to train a support vector machine based on 78 different input parameters. For input parameters we used the original image, a BM3D filtered image [30] and a k-means classification identifying background (not used), medium bright and bright pixels [27]. Using the BM3D filtered and original image we applied a maximum, minimum, standard deviation, entropy, range and band pass filter with 5 different filter sizes (3, 5, 9, 15, 21 pixels), as well as an anisotropy filter [28] with 5 different Gaussian filter sizes and standard deviations (size = [0.6,1.2,3,6,12] pixel and deviation = [0.2, 0.4, 1, 2, 4] pixel), to account for the different size of the actin structures. This input data was analyzed using a principle component analysis (PCA) to extract the principle components accounting for 95% of the variance, to reduce computational complexity. The obtained principle components were than used for training of the SVM. Subsequent application of the trained SVM to the remaining images allowed measuring the percentage of the 4 types of actin structures.

### RNA extraction and real-time quantitative reverse transcriptase polymerase chain reaction (qRT-PCR)

For expression analysis, total RNA was isolated from harvested cells using the Universal RNA Purification Kit (Eurex®, Gdansk, Poland) according to manufacturer’s instructions. RNA was quantified with Nanodrop (Peqlab, Erlangen, Germany), and 50 ng of total RNA was reverse transcribed utilizing 2.5 μM random hexamers (Invitrogen) in a reaction mix containing 5 mM MgCl_2_ (ABI Applied Biosystems, Foster City, CA, USA), 1x reaction buffer (ABI Applied Biosystems), 4 mM pooled dNTPs (Roboklon, Berlin, Germany), 1 U/μl RNAse inhibitor (Thermo Fisher) and 2.5 U/μl Moloney Murine Leukemia Virus reverse transcriptase (Thermo Fisher). The RNA complemented mix was incubated at 25 °C for 5 min, 42 °C for 45 min, 95 °C for 5 min with subsequently cooling at 4 °C. The cDNA products were amplified using SYBR Green dye chemistry and the LightCycler 480 (Roche Diagnostics, Basel, Switzerland) under the following conditions: 95 °C for 2 min followed by 45 cycles of 95 °C for 7 s, 60 °C for 7 s, 60 °C for 10 s and 72 °C for 20 s. The used primers are listed in Table 1.

**Table 1:** Oligonucleotide sequences used for qPCR

Oligonucleotide sequences	Strand specificity	Sequence (5′ – 3′)	Amplicon length
*CD44*	forward	CTG GCG CAG ATC GAT TTG AA	244 bp
	reverse	TTG CTG CAC AGA TGG AGT TGG	
*CDH2*	forward	TGG GAA TCC GAC GAA TGG	65 bp
	reverse	TGC AGA TCG GAC CGG ATA CT	
*FN1*	forward	GGA GTT GAT TAT ACC ATC ACT G	259 bp
	reverse	TTT CTG TTT GAT CTG GAC CT	
*G6PDH*	forward	GAA GAT GGT GAT GGG ATT TC	113 bp
	reverse	GAA GGT GAA GGT CGG AGT	
*ITGA5*	forward	TGC CTC CCT CAC CAT CTT C	171 bp
	reverse	TGC TTC TGC CAG TCC AGC	
*ITGB1*	forward	CAA AGG AAC AGC AGA GAA GC	168 bp
	reverse	ATT GAG TAA GAC AGG TCC ATA AGG	
*LAMR1*	forward	GCC ATT GAA AAC CCT GCT AG	242 bp
	reverse	AGC GCA ATG GTA GGT AGG TT	
*MACC1*	forward	TTC TTT TGA TTC CTC CGG TGA	136 bp
	reverse	ACT CTG ATG GGC ATG TGC TG	

Data analysis was performed with LightCycler 480 Software release 1.5.1 (Roche Diagnostics). Mean values were calculated from duplicate qPCR reactions. Each mean value of the various genes was normalized to the appropriate mean cDNA amount of the housekeeping gene glucose-6-phosphate dehydrogenase (G6PDH). Oligonucleotide sequences used can be found in Table 1.

### Statistics

Statistics was performed using the two-sided Mann-Whitney-Wilcoxon test or Kruskal-Wallis test with Tukey post-hoc test. Significance was defined for *p* < 0.05. All error-bars depict the standard error of the mean. Experiments were repeated at least three independent times.

## Results

### MACC1 increases single cell motility and elastic modulus

**Fig. 1:**
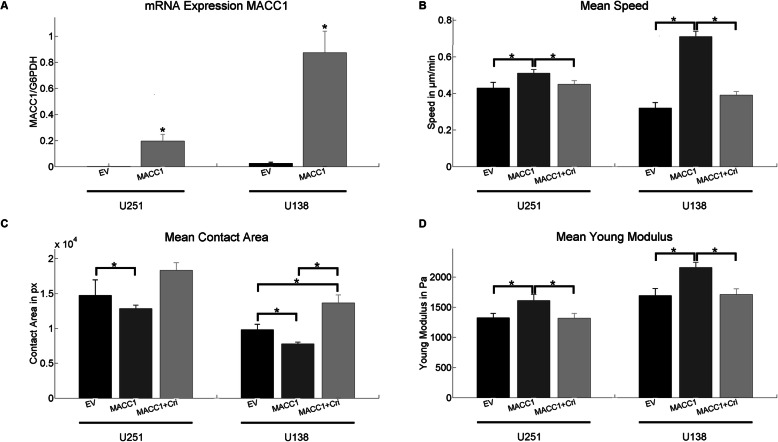
MACC1 increases cell motility and the elastic modulus, and reduces the cell-surface contact. **a** Verification of MACC1 overexpression in U251/MACC1 and U138/MACC1 cells. **b** MACC1 overexpression led to a higher motility in U251 and U138 cells, which could be inhibited by crizotinib. **c** MACC1 overexpression led to a lower contact area of both U251 and U138 cells. Crizotinib application reversed the effect leading to an increase in contact area. The following numbers of cells were measured in three independent experiments (**b** and **c**): n_U138/EV_ = 67, n_U138/MACC1_ = 164, n_U138/MACC1 + Cri_ = 102, n_U251/EV_ = 51, n_U251/MACC1_ = 51 and n_U251/MACC1 + Cri_ = 45. D) MACC1 overexpression resulted in an increase of the Young’s modulus in U251 and U138 cells, which could be inhibited by crizotinib. The following numbers of cells were measured in three independent experiments: n_U138/EV_ = 60, n_U138/MACC1_ = 85, n_U138/MACC1 + Cri_ = 88, n_U251/EV_ = 34, n_U251/MACC1_ = 32 and n_U251/MACC1 + Cri_ = 35. Stars depict *p* < 0.05. Error bars depict the standard error of the mean

We first evaluated if *MACC1* overexpressed in the GBM cell lines U251 and U138 (Fig. 1a). *MACC1* levels are associated with changes in the migratory behavior of isolated, single cells. We observed an increase in mean speed for both U251/MACC1 (v _U251/EV_ = 0.43 μm/min, v _U251/MACC1_ = 0.51 μm/min) and U138/MACC1 (v _U138/EV_ = 0.32 μm/min, v _U138/MACC1_ = 0.71 μm/min) cells, relative to their respective controls (Fig. 1b). This effect was attenuated after the application of crizotinib (v _U251/MACC1 + Cri_ = 0.45 μm/min, v _U138/MACC1 + Cri_ = 0.39 μm/min), an inhibitor of the transcriptional *MACC1* target c-Met (Fig. 1b). Additionally, the contact area of single cells with the substrate was reduced in *MACC1* overexpressing cells (A _U251/EV_ = 14,740 px, A _U251/MACC1_ = 12,830 px, A _U138/EV_ = 9809 px, A _U138/MACC1_ = 7776 px). This effect was fully inhibited by crizotinib in U251/MACC1 (A _U251/MACC1 + Cri_ = 18,338 px) and U138/MACC1 (A _U138/MACC1 + Cri_ = 13,657 px) cells (Fig. 1c). Notably, the contact area of U138/MACC1 cells exceeded control levels after crizotinib application (Fig. 1c). Faster migration and less contact area of *MACC1*-overexpressing GBM cells point to possible changes in their mechanical or adhesive properties. Evaluating the mechanical properties of single GBM cells, an increased *MACC1* expression was associated with an increased resistance to deformation (Young’s modulus) of U251 (E _U251/EV_ = 1.33 kPa, E _U251/MACC1_ = 1.61 kPa) and U138 (E _U138/EV_ = 1.69 kPa, E _U138/MACC1_ = 2.16 kPa) cells. The effect was reversed by the c-Met inhibitor crizotinib (E _U251/MACC1 + Cri_ = 1.32 kPa, E _U138/MACC1 + Cri_ = 1.71 kPa) (Fig. 1d).

### MACC1 inhibits the aggregation of 3D tumor aggregates and lowers cell-cell-adhesion

**Fig. 2:**
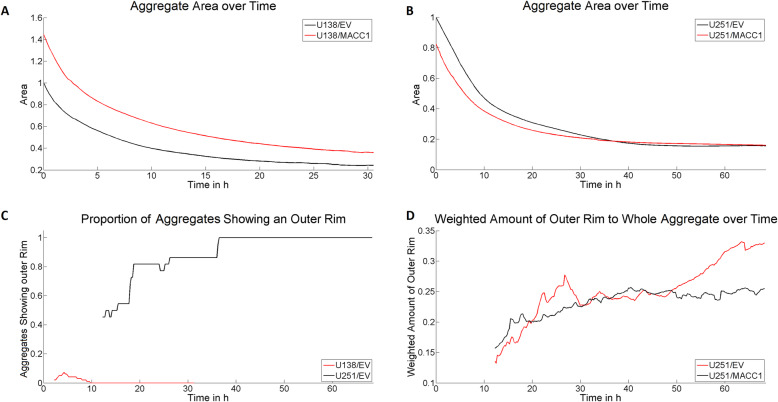
MACC1 influences the formation and structure of 3D aggregate formation. **a**, **b** MACC1 dependent size measurement of U138 and U251 3D aggregates. While MACC1 overexpression in U138 cells led to larger aggregates, this effect was not visible in U251 spheroids. **c** Evaluation of the occurrence of an “outer rim” in U251 and U138 3D aggregates. These structures are relevant for U251 aggregates only. **d** Proportion of the “outer rim” relative to the whole 3D aggregates of U251/EV and U251/MACC1 cells. MACC1 overexpression led to a reduced formation of dense ring structures. The following numbers of 3D aggregates were measured in three independent experiments: n_U138/EV_ = 99, n_U138/MACC1_ = 46, n_U251/EV_ = 22 and n_U251/MACC1_ = 52

Both migratory and mechanical properties are linked to intra-cellular force generation, adhesion and/or surface tension, and thus with formation of 3D aggregates [31, 32]. Consequently, we evaluated whether *MACC1* overexpression impacts 3D aggregate formation by determining their size, shape and compactness over time. Measurement of U138 cells had to be limited to 32 h because of bursting of aggregates, emitting necrotic material (Additional file 2). In general, U251 cells formed larger, less compact and slightly more circular 3D aggregates compared to U138 cells (Additional file 3, Additional file 4, Additional file 5). Furthermore, U251 cells showed a different aggregation behavior than U138 cells. While U138 cells formed compact structures within the whole 3D aggregate, U251 cells aggregated in an outside-in manner (Additional file 1 A, Additional file 4, Additional file 5). No significant changes of the average compactness and shape of both U251/MACC1 and U138/MACC1 aggregates, compared to their controls, were found (Additional file 6). In contrast, the size of aggregates formed by U138/MACC1 cells were 1.4–1.5 times larger than those of U138/EV cells (Fig. 2a) whereas U251/MACC1 cells showed no increase in aggregate size compared to U251/EV cells (Fig. 2b). Interestingly, U251 aggregates formed a compact “outer rim” structure that was missing in U138 aggregates (Additional file 1 A, Fig. 2c-d). Analyzing the proportion of the “outer rim” in relation to the whole 3D aggregate on longer time scales (> 50 h), the overexpression of *MACC1* was associated with a less pronounced ring structure (0.75 fold proportion, Fig. 2d, Additional file 7). In summary, high *MACC1* expression hinders the aggregation not only in U138 cells but also the formation of compact three dimensional structures in U251 cells. With the observed parameters for the formation of 3D aggregates, we were able to estimate a 12% higher cell-cell adhesion for U138/EV than for U138/MACC1 and 42% higher cell-cell adhesion for U251/EV than U251/MACC1 GBM cells.

### MACC1 does not change distribution and mRNA expression of several key adhesion molecules nor cell-ECM adhesion

**Fig. 3:**
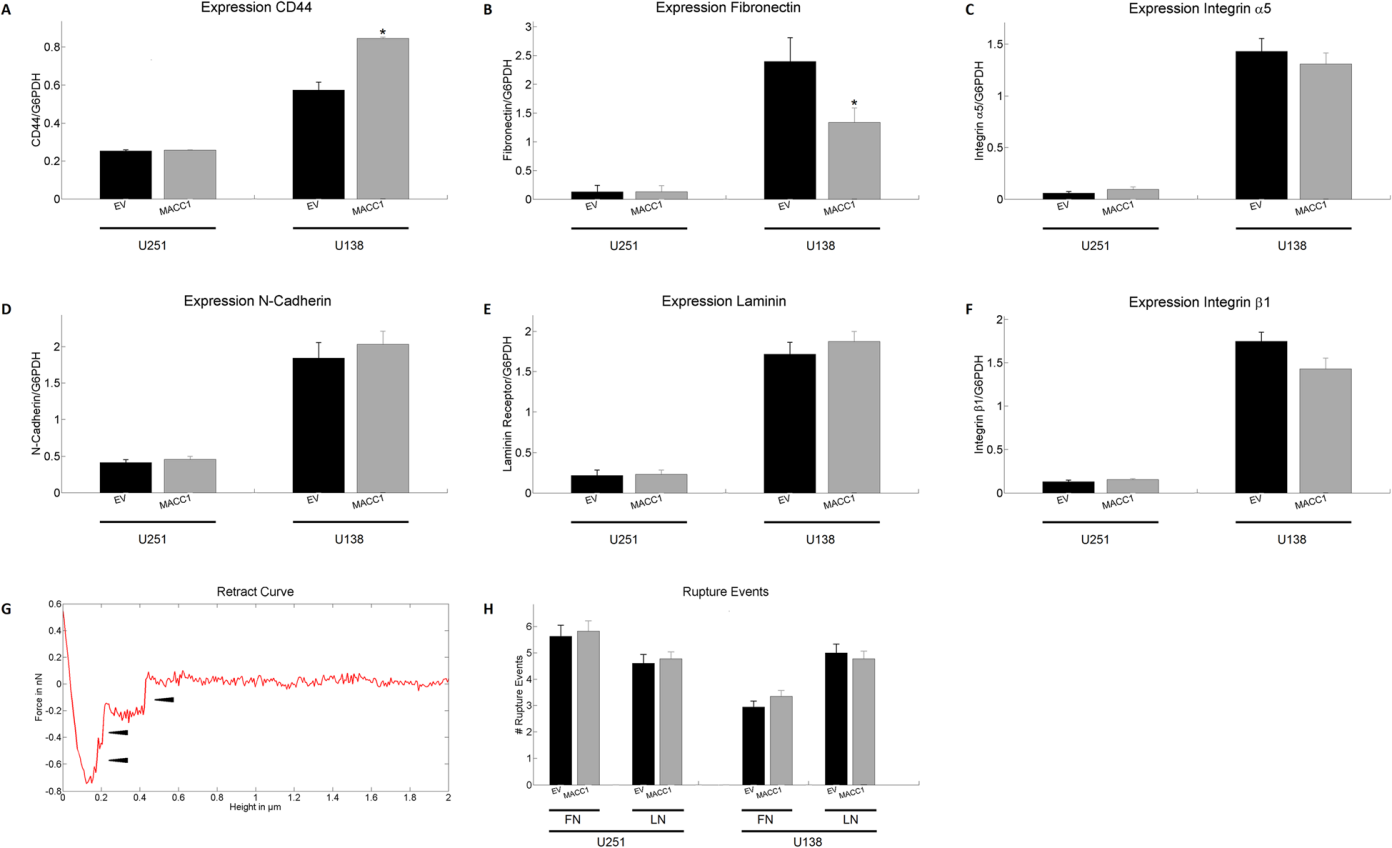
Effect of MACC1 overexpression on adhesion. **a**-**f** mRNA expression of CD44, fibronectin, integrin α5, β1, N-cadherin, laminin receptor 1 of U251 and U138 cells with and without overexpression of MACC1. No consistent MACC1 associated effect was observed. *n* = 3. **g** Illustration of a typical retract curve when using a coated cantilever. Arrowheads show discrete rupture events used for quantification. **h** Mean number of discrete adhesion events between U251 or U138 cells and laminin or fibronectin coated cantilevers. No MACC1 dependent effect was observed. The following numbers of cells were measured in three independent experiments: n_U138/EV FN_ = 40, n_U138/EV LN_ = 35, n_U138/MACC1 FN_ = 40, n_U138/MACC1 LN_ = 35, n_U251/EV FN_ = 30, n_U251/EV LN_ = 33, n_U251/MACC1 FN_ = 35 and n_U251/MACC1 LN_ = 35. Stars depict p < 0.05. Error bars depict the standard error of the mean

An important key parameter influencing both cell motility and contact area to the substrate is cell adhesion. We next compared the mRNA expression of the adhesion molecules CD44, N-cadherin, integrin α5 and β1, laminin (LN) receptor 1, as well as the expression of the extracellular matrix molecule fibronectin (FN). Notably, U251 cells showed for all mRNA significantly lower expression levels than U138. An increased expression of CD44 (1.5 fold) and a reduced expression of fibronectin (0.6 fold) were observed in *MACC1* overexpressing U138 (Fig. 3a and b). The other analyzed factors displayed no significant changes (Fig. 3a-f).

To further assess whether *MACC1* alters the affinity of GBM cells to specific substrates we indented these cells with a cantilever coated either with FN or LN. The resulting retraction curves showed discrete rupture events (Fig. 3g), being a measure for short term adhesion to FN or LN. *MACC1* overexpression in both U251 and U138 cells did not significantly alter the short term adhesion to FN or LN on the time scale of 30 s (Fig. 3h).

**Fig. 4:**
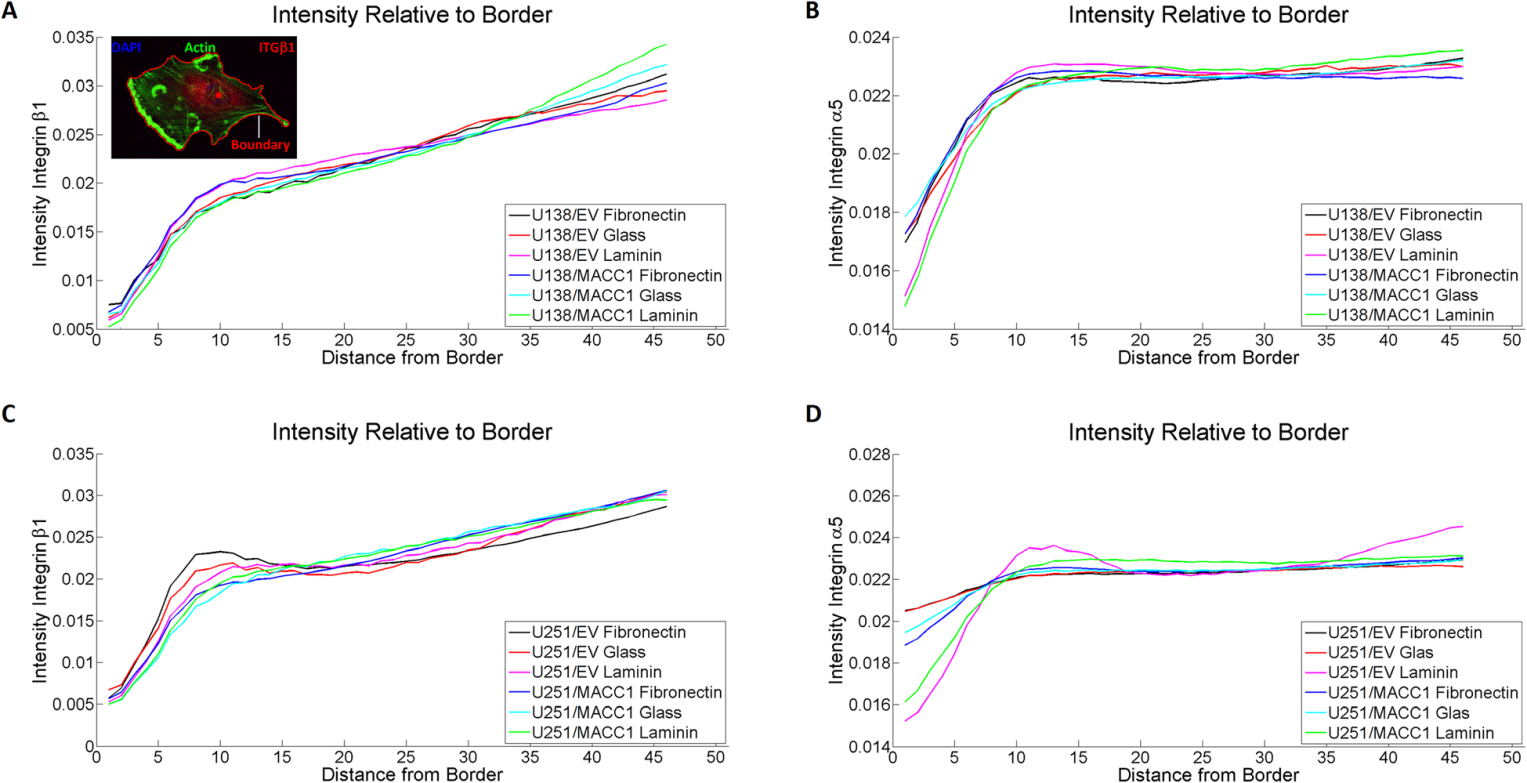
MACC1 does not affect integrin distribution. **a**, **b** Distribution of integrin β1 and α5 relative to the cell boundary for U138/EV and U138/MACC1 cells, plated on glass, fibronectin and laminin. No significant change in distribution could be observed. The inlet in **a**) displays an U138 cell labeled with DAPI (nucleus), phalloidin (actin) and an integrin β1 antibody. The red closed line shows the boundary and the red dot in the cell depicts the center of the nucleus. **c**, **d** Distribution of integrin β1 and α5 relative to the cell boundary for U251/EV and U251/MACC1 cells, plated on glass, fibronectin and laminin. No significant change in distribution could be observed. The following numbers of images of three independent experiments were analyzed: n_U138/EV Glass_ = 17, n_U138/EV FN_ = 34, n_U138/EV LN_ = 52, n_U138/MACC1 Glass_ = 30, n_U138/MACC1 FN_ = 44, n_U138/MACC1 LN_ = 38, n_U251/EV Glass_ = 35, n_U251/EV FN_ = 67, n_U251/EV LN_ = 70, n_U251/MACC1 Glass_ = 36, n_U251/MACC1 FN_ = 77 and n_U251/MACC1 LN_ = 55

Another possible mechanism for *MACC1* to influence adhesion is via changes in the intra-cellular distribution of adhesion molecules. Thus, the relationship between *MACC1*-overexpression and localization of integrin α5 and β1 was determined by analyzing the distribution of the two molecules relative to the cell boundary for cells plated on glass, FN and LN (Fig. 4a, inlet). Successful coating was examined using immunofluorescence staining (Additional file 8). The distribution of integrin α5 and β1 on all three substrates showed no alterations induced by *MACC1*-overexpression in U251 and U138 cells (Fig. 4a-d). Similarly, the integrin distribution of cells in the process of adhesion (30 min after seeding) was not altered, supporting the previous findings (Additional file 9, Additional file 10).

### MACC1 increases protrusive actin in GBM cells

The observed *MACC1*-dependent effects point towards an involvement of the cytoskeleton. We found the intermediate filament GFAP expressed in both U251 and U138 cells, organized in a mostly perinuclear localization, and absent in the cell periphery. *MACC1* expression did not influence GFAP organization in both cell lines (Additional file 11). Vimentin was also localized perinuclearly in both cell lines. Interestingly, vimentin was observed in the periphery of U251 cells, but not in U138 cells. The overexpression of *MACC1* did not alter the organization of the vimentin cytoskeleton in both GBM cell lines (Additional file 12). The organization of microtubules, around the nucleus, with extensions into the periphery, was not influenced by *MACC1*-overexpression (Additional file 13).

**Fig. 5:**
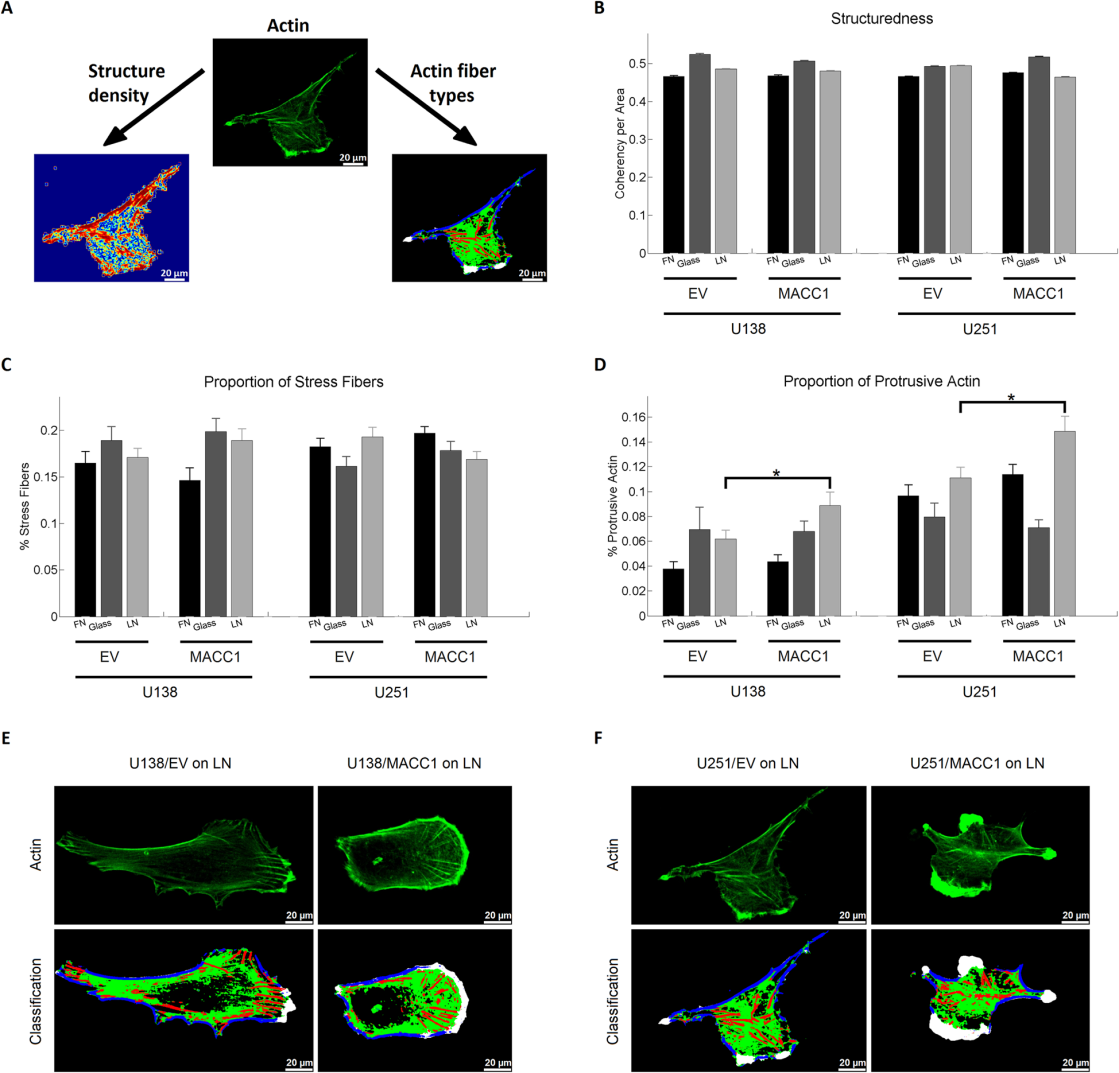
MACC1 increases the amount of protrusive actin. **a** Illustration of the two different approaches of the actin structure analysis, including the calculation of the structure density (bottom left) and classification of different actin structures (bottom right). Stress fibers are depicted in red, cortical fibers in blue, protrusive actin in white and punctuate actin in green. **b** Results of the structure density analysis. MACC1 overexpression did not have a significant effect on the structuredness of the actin cytoskeleton of U138 and U251 cells on neither substrate. **c**, **d** Results of actin fiber type analysis. MACC1 increased the amount of protrusive actin for U138 and U251 cells on laminin. **e** and **f** Representative images of U138 and U251 cells on laminin, illustrating the increase of protrusive actin for MACC1-overexpressing cells. The following numbers of images of three independent experiments were analyzed: n_U138/EV Glass_ = 17, n_U138/EV FN_ = 34, n_U138/EV LN_ = 52, n_U138/MACC1 Glass_ = 30, n_U138/MACC1 FN_ = 44, n_U138/MACC1 LN_ = 38, n_U251/EV Glass_ = 35, n_U251/EV FN_ = 67, n_U251/EV LN_ = 70, n_U251/MACC1 Glass_ = 36, n_U251/MACC1 FN_ = 77 and n_U251/MACC1 LN_ = 55. Stars depict p < 0.05. Error bars show the standard error of the mean. Scale bars depict 20 μm

Next, we measured *MACC1*-dependent actin cytoskeleton organization in terms of structure density and type of actin fibers formed (Fig. 5a). Analyzing the structuredness of the actin cytoskeleton in cells attached to glass, FN or LN, no association of the structure density was found with *MACC1* expression (Fig. 5b). To determine the organization of the actin cytoskeleton in more detail we identified 4 different types of actin structures (Fig. 5a), using self-developed software based on support vector machines: stress fibers (red), cortical fibers (blue), protrusive actin (white) and punctuate actin (green). *MACC1* overexpression did not change the amount of stress fibers (Fig. 5c), but resulted in an accumulation of protrusive actin in U138/MACC1 (1.45 fold) and U251/MACC1 (1.38 fold) cells on LN, but neither on glass nor FN (Fig. 5d-f).

## Discussion

In this study we report the first biomechanical effects induced by *MACC1*. We demonstrated that *MACC1* overexpression causes an increase in single cell speed and elastic modulus of GBM cells in a c-Met-dependent manner. These effects are at least in part caused by substrate dependent changes in the actin cytoskeleton organization. We furthermore found *MACC1* to inhibit 3D aggregation dynamics of GBM cells, caused by an increased elastic modulus and reduced cell-cell-adhesion.

GBM is a mostly lethal tumor entity, with median survival of 14 month. The infaust prognosis is due to the almost inevitable recurrence, caused by the diffuse migration pattern of tumor cells and intratumoral heterogeneity [3, 33]. *MACC1* was previously shown to increase migration in many tumor types, including GBM [13, 16]. There, migration of GBM cells was measured in a Boyden chamber assay with impedance as read-out. Migration was assessed over the time course of 40 h, thus a mixture of multiple parameters including migration, elastic moduli and proliferation was assessed. Here, we verified previous results demonstrating, that *MACC1* overexpression is indeed associated with increased migration on a single cell level in the two GBM cell lines used [16]. The behavior was accompanied by a lower contact area between cells and substrate. Both migration and contact area pointed towards changes in adhesion, contractility, elasticity and/or surface tension [34, 35].

Subsequent measurements of the Young’s modulus demonstrated that *MACC1* was associated with increased elasticity. This generally results in a moderately reduced migrational capacity as forces necessary to deform the cell for movement have to be higher and thus contrasting the increased migration observed [34, 36]. A recent study identified microRNA-598 to inhibit *MACC1* and c-Met/Akt signaling in GBM [17]. Although the direct activation of c-Met/Akt via *MACC1* was not addressed in that study, it is regulated by *MACC1* in many tumor types [13, 16]. As c-Met signaling can induce migration, we tested if the c-Met inhibitor crizotinib might modulate *MACC1*-associated effects [7]. All effects regarding cell speed, contact area and elastic modulus were abrogated after crizotinib treatment, pointing to c-Met as regulator of *MACC1*-induced migration and increase of elastic modulus. HGF/c-Met signaling axis leads to phosphorylation of the membrane-actin cortex linker ezrin [7]. Activation of ezrin and subsequent stabilization of the actin cortex is a possible explanation for the increased Young’s modulus measured here [37]. As indentations in our experiments were in the order of 0.5–0.8 μm we expected our measurements to mainly reflect actin cortex properties and thus membrane-actin cortex linker proteins to play a major role [38, 39].

Consequently, we next checked key adhesion molecules affected by *MACC1*, because a reduced adhesion may be associated with reduced contact area and altered migratory capacity, counteracting the effects of the increased elastic modulus [40, 41]. We found no significant changes in the expression, distribution and affinity to the analyzed adhesion molecules. To the best of our knowledge there is no other study that determined a *MACC1* dependent distribution of adhesion molecules or to factors associated with adhesion. So far, only MACC1-dependent expression of adhesion molecules, but not distribution, was assessed in non-brain tumors [10, 42–46]. These studies were reporting a positive correlation between expression of *MACC1* and FN, CD44 and N-cadherin and a negative correlation with E-cadherin expression [10, 42–46]. These findings were discussed in the context of *MACC1*-induced epithelial-to-mesenchymal transition (EMT), but the concept of EMT cannot be fully transferred to GBM [47].

Previous studies performed in GBM and other tumor types found a *MACC1* dependent regulation of PI3K and c-Met [10–13, 16]. Both molecules are associated with a reorganization of the cytoskeleton [7–9], rendering actin, intermediate filaments and microtubules plausible targets to explain MACC1-dependent effects on migration and elastic modulus [41]. Thus, we evaluated *MACC1*-dependent effects on cytoskeletal organization. We did not find any changes in the organization of vimentin, GFAP and microtubules, but a LN dependent increase in the formation of protrusive actin in *MACC1*-overexpressing cells. In agreement, MACC1 expression was found to be associated with increased actin fluorescence intensity in HeLa cells [48]. Our finding agrees with the general observation that GBM cells invade mainly along white matter tracts and blood vessels [49]. Consequently, one potential mechanism explaining increased *MACC1* dependent brain infiltration is the contact of these cells with the basement membrane and thus LN-induced stronger actin polymerization at the cell front, subsequently facilitating migration. On the molecular level *MACC1* might induce an activation of c-Met causing an activation of RAP1 [50], resulting in an activation of integrins (inside-out signaling) and thus a subsequent outside-in signaling leading to generation of protrusive actin [51]. A similar mechanism induced by *MACC1* induced PI3K activation seems also possible [13, 52]. As we did not observe a *MACC1*-dependent increase in protrusive actin on FN, it excludes all integrins binding to both FN and LN, leaving integrins α6, α3 and β4 as potential targets [53, 54]. A previous study on glioma migration along linear laminin tracks demonstrated glioma migration to be dependent on formins, being – together with the Arp2/3 complex – potential nucleation factors of protrusive actin, as both are activated via integrin binding [41, 55]. Nevertheless, different mechanisms might also be responsible. According to Gritsenko and Friedl, glioblastoma migration on laminin can only be partly inhibited by combined blockade of integrins β1, αV and α6β4, proposing additional integrin independent mechanisms [40].

To further validate if MACC1 overexpression alters cell-cell interactions we analyzed 3D tumor aggregation to proof the significance of MACC1 overexpression in our system. Both cell lines differed in the speed and type of aggregation. Aggregation depends on adhesion, contractility and surface tension, being in agreement with the observed lower expression of mRNA for adhesion molecules in U251 cells and their slower aggregation compared to U138 cells [25, 56]. This might also cause different modes of aggregation, as it is energetically less favorable for U251 cells in the spheroid center to form cell-cell contacts, while cells in the boundary region have a larger medium-cell interface favoring adhesion and thus formation of 3D structures.

From aggregation experiments, we directly demonstrated for the first time that *MACC1*-overexpressing cells are less adhesive causing a reduction in 3D aggregation. Additionally, the increased elastic modulus of *MACC1*-overexpressing cells counteracts or limits the increase in contact area of neighboring cells induced by cell-cell adhesion and thus further impedes 3D aggregation [57]. Consequently, *MACC1* impedes 3D aggregation via both a lower adhesion and an increased cortical elasticity.

## Conclusions

In this work we demonstrate that *MACC1* increases the migration speed of single cells and their elastic modulus. Furthermore, *MACC1* inhibits the speed of spontaneous aggregation. These effects depend on reduced adhesion, increased cortical elasticity and elevated amounts of protrusive actin. In turn, targeting *MACC1* expression and c-Met signaling might inhibit GBM cell migration and thus improve outcome for patients. This possible treatment option warrants further investigation.

## Supplementary information

**Additional file 1: Figure S1**. Illustration of local 3D aggregate analysis. A, B) Sample 3D aggregates of U251 and U138 cells after approximately 24 h of aggregation time. C) Intensity distribution as a function of distance to the center of the 3D aggregates in A and B normalized to the background.

**Additional file 2: Vid S1**. U138/EV 3D aggregate displaying bursting near the end of the measurement time.

**Additional file 3: Figure S2**. Measurement of size, optical density and shape of U138/EV and U251/EV 3D aggregates over time. A) Depicts the 3D aggregate size of U251 and U138 over time. U251 aggregates remain significantly larger. B) Shows the 3D aggregate compactness of U251 and U138 over time. U251 aggregates stayed less compact. D) Illustrates the aggregates circularity of U251 and U138 over time. U251 aggregates are slightly more circular than U138 aggregates. The following numbers of 3D aggregates were measured in three independent experiments: n_U138/EV_ = 99 and n_U251/EV_ = 22.

**Additional file 4: Vid S2**. Typical U138 aggregation sequence.

**Additional file 5: Vid S3**. Typical U251 aggregation sequence.

**Additional file 6: Figure S3**. Measurement of *MACC1* dependence of optical density and shape of U138 and U251 3D aggregates over time. A, B) Depicts the 3D aggregate compactness and C, D) the circularity of U251 and U138 with and without *MACC1* overexpression over time. No significant *MACC1* associated differences could be observed. The following numbers of 3D aggregates were measured in three independent experiments: n_U138/EV_ = 99, n_U138/MACC1_ = 46, n_U251/EV_ = 22 and n_U251/MACC1_ = 52.

**Additional file 7: Vid S4**. Comparison of U251/EV and U251/MACC1 3D aggregation. Denote the larger fraction of the outer rim in U251/EV cells at the end of imaging process.

**Additional file 8: Figure S4**. Validation of the fibronectin and laminin coating. The left column shows the negative control, treated identically to the coated ones, except for the application of fibronectin or laminin. The right column shows the respective fibronectin or laminin coating. One can see that the coating could be verified.

**Additional file 9: Figure S5**. Integrin α5 and β1 distribution on FN for cells allowed to adhere for 30 min. Integrins were mainly localized near the nucleus or the expanding actin cytoskeleton. No significant *MACC1*-dependent change in integrin distribution could be observed. *n* > 9. Scale bar depicts 50 μm.

**Additional file 10: Figure S6**. Integrin α5 and β1 distribution on LN for cells allowed to adhere for 30 min. Integrins were mainly localized near the nucleus or the expanding actin cytoskeleton. No significant *MACC1*-dependent change in integrin distribution could be observed. n > 9. Scale bar depicts 50 μm.

**Additional file 11: Figure S7**. Staining of U138 and U251 cells for GFAP. *MACC1* overexpression was not associated with a visible change in GFAP organization. Scale bar corresponds to 25 μm.

**Additional file 12: Figure S8**. Staining of U138 and U251 cells for vimentin. *MACC1* overexpression was not associated with a visible change in vimentin organization. Scale bar corresponds to 25 μm.

**Additional file 13: Figure S9**. Staining of U138 and U251 cells for βIII-tubulin. *MACC1* overexpression was not associated with a visible change in microtubule organization. Scale bar corresponds to 25 μm.

**Additional file 14: Supplemental Results**. Estimation of cell-cell adhesion from 3D aggregate formation.

## Data Availability

The datasets used and/or analyzed during the current study are available from the corresponding author on reasonable request.

## Abbreviations

AFM: Atomic force microscopy
DAPI: 4′,6-Diamin-2-phenylindol
EMT: Epithelial-to-mesenchymal transition
FN: Fibronectin
GBM: Glioblastoma
GFAP: Glial fibrillary acidic protein
HGF: Hepatocyte growth factor
LN: Laminin
MACC1: Metastasis associated in colon cancer 1
PCA: Principal component analysis
SVM: Support vector machine
