# Supplementary figures and images for: MACC1 driven alterations in cellular biomechanics facilitate cell motility in glioblastoma
Source: Cell Commun Signal. 2020 Jun 5;18:85. doi: 10.1186/s12964-020-00566-1 (PMC7275321; doi:10.1186/s12964-020-00566-1)

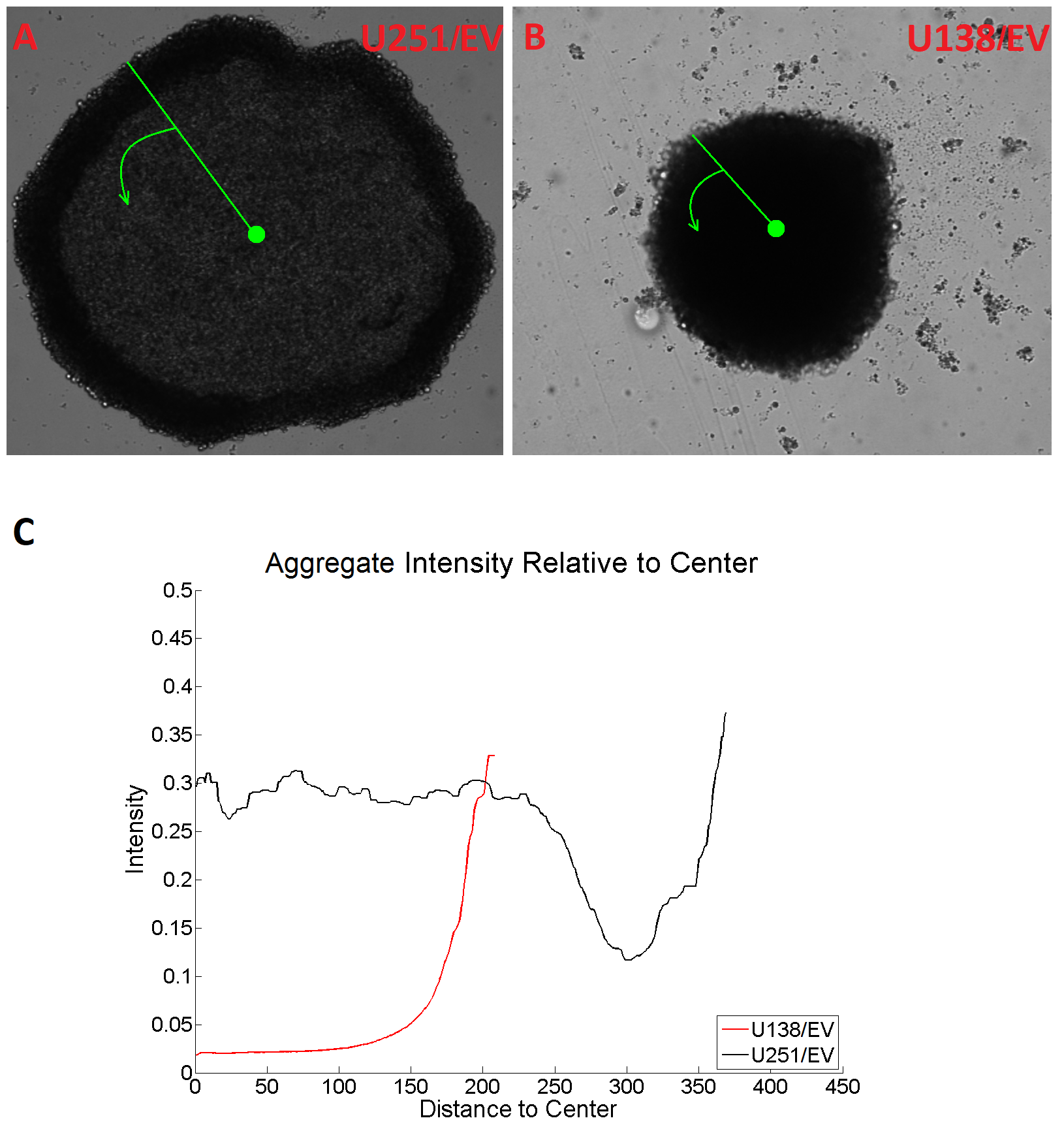

Supplement: Supplementary file 2 — Additional file 1: Figure S1. Illustration of local 3D aggregate analysis. A, B) Sample 3D aggregates of U251 and U138 cells after approximately 24 h of aggregation time. C) Intensity distribution as a function of distance to the center of the 3D aggregates in A and B normalized to the background. [file 12964_2020_566_MOESM2_ESM.tif]

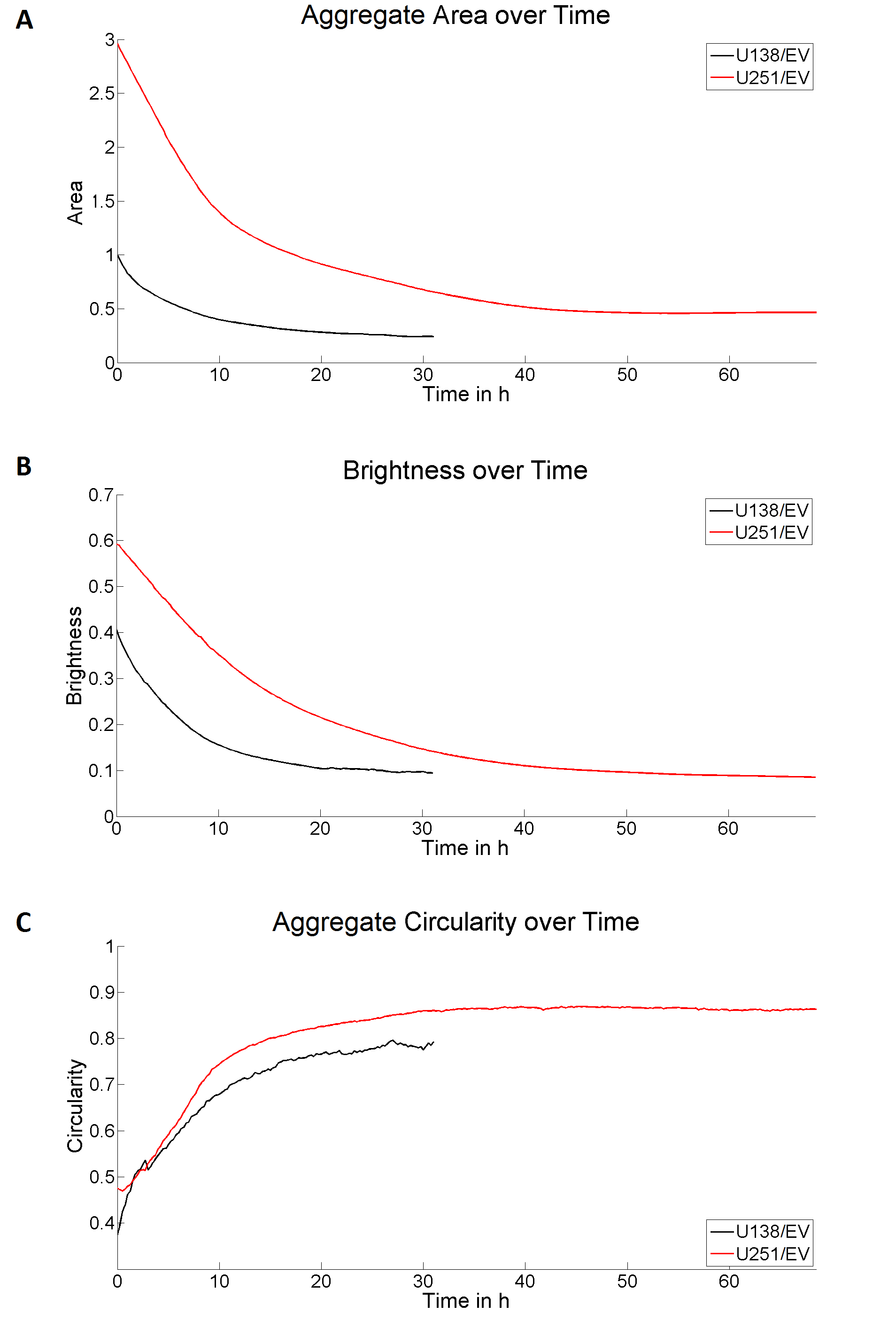

Supplement: Supplementary file 4 — Additional file 3: Figure S2. Measurement of size, optical density and shape of U138/EV and U251/EV 3D aggregates over time. A) Depicts the 3D aggregate size of U251 and U138 over time. U251 aggregates remain significantly larger. B) Shows the 3D aggregate compactness of U251 and U138 over time. U251 aggregates stayed less compact. D) Illustrates the aggregates circularity of U251 and U138 over time. U251 aggregates are slightly more circular than U138 aggregates. The following numbers of 3D aggregates were measured in three independent experiments: nU138/EV = 99 and nU251/EV = 22. [file 12964_2020_566_MOESM4_ESM.tif]

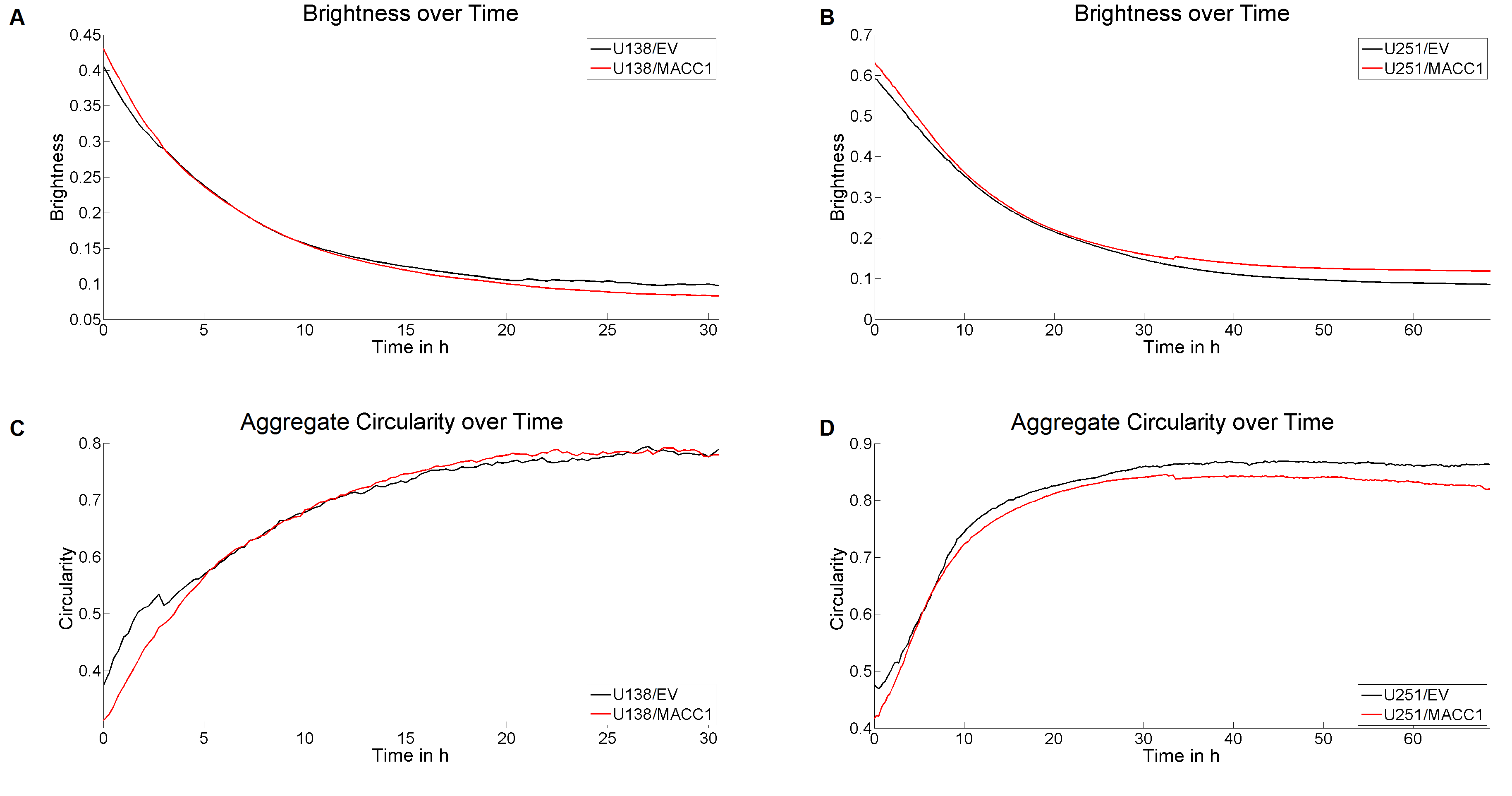

Supplement: Supplementary file 7 — Additional file 6: Figure S3. Measurement of MACC1 dependence of optical density and shape of U138 and U251 3D aggregates over time. A, B) Depicts the 3D aggregate compactness and C, D) the circularity of U251 and U138 with and without MACC1 overexpression over time. No significant MACC1 associated differences could be observed. The following numbers of 3D aggregates were measured in three independent experiments: nU138/EV = 99, nU138/MACC1 = 46, nU251/EV = 22 and nU251/MACC1 = 52. [file 12964_2020_566_MOESM7_ESM.tif]

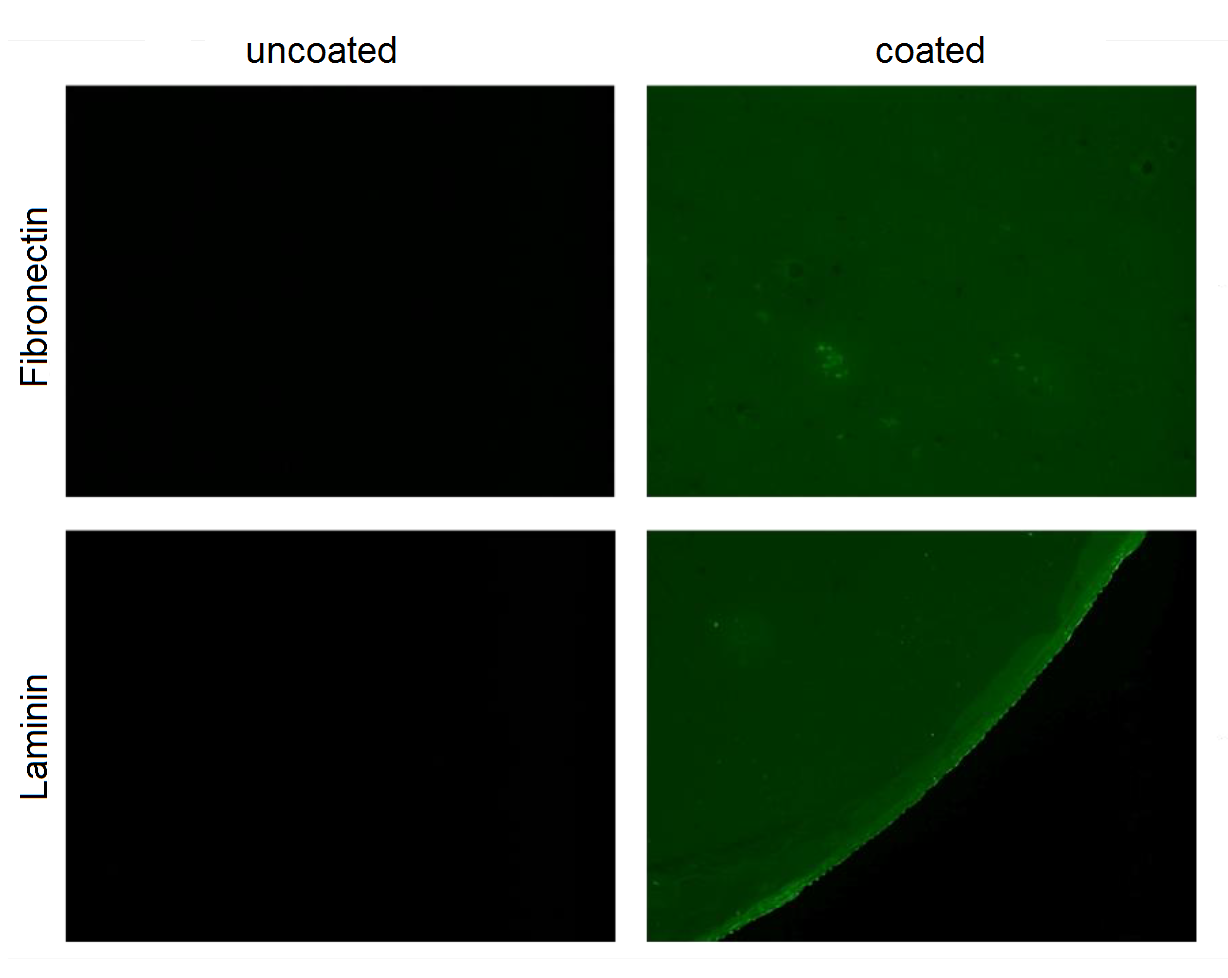

Supplement: Supplementary file 9 — Additional file 8: Figure S4. Validation of the fibronectin and laminin coating. The left column shows the negative control, treated identically to the coated ones, except for the application of fibronectin or laminin. The right column shows the respective fibronectin or laminin coating. One can see that the coating could be verified. [file 12964_2020_566_MOESM9_ESM.tif]

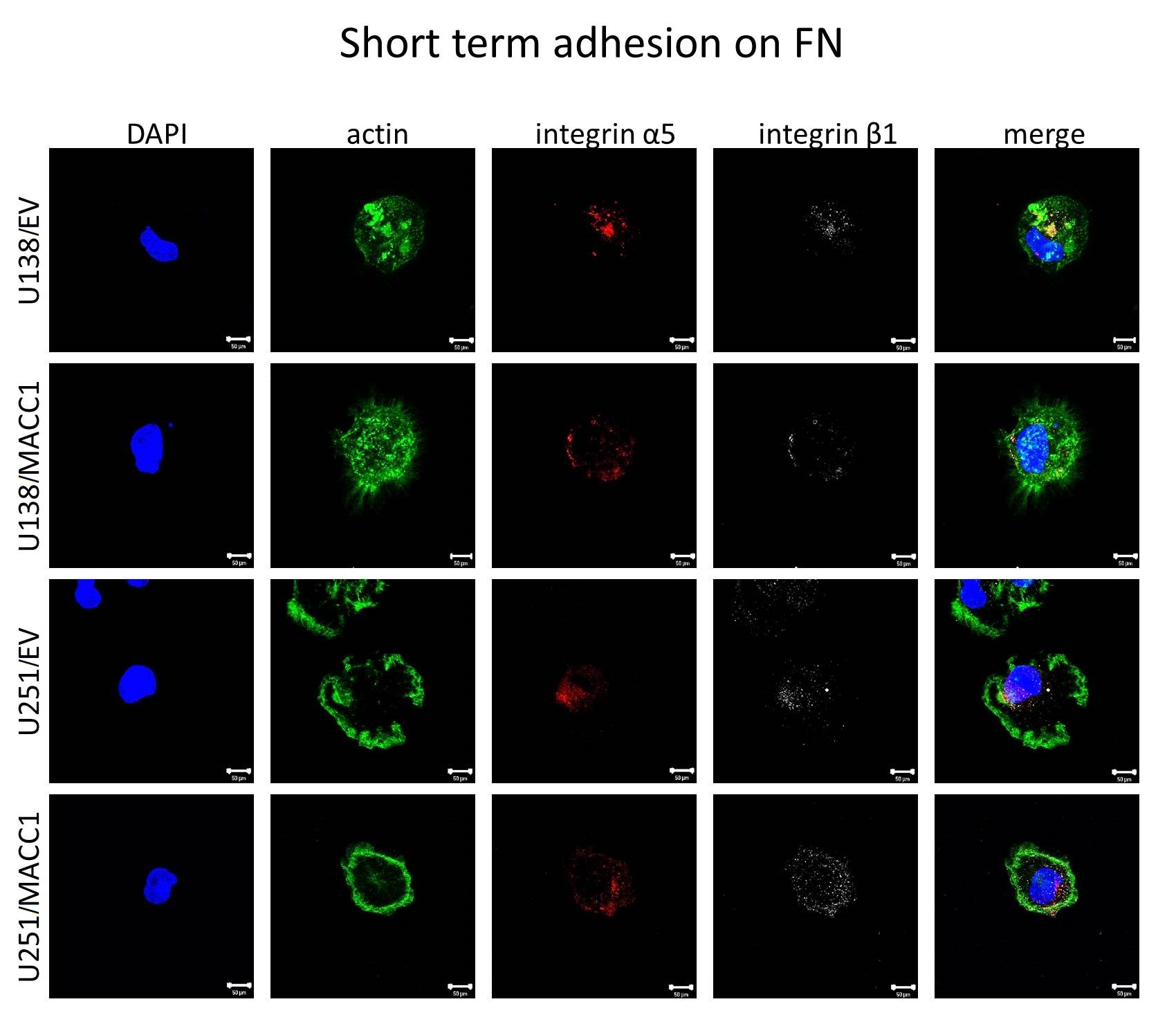

Supplement: Supplementary file 10 — Additional file 9: Figure S5. Integrin α5 and β1 distribution on FN for cells allowed to adhere for 30 min. Integrins were mainly localized near the nucleus or the expanding actin cytoskeleton. No significant MACC1-dependent change in integrin distribution could be observed. n > 9. Scale bar depicts 50 μm. [file 12964_2020_566_MOESM10_ESM.tif]

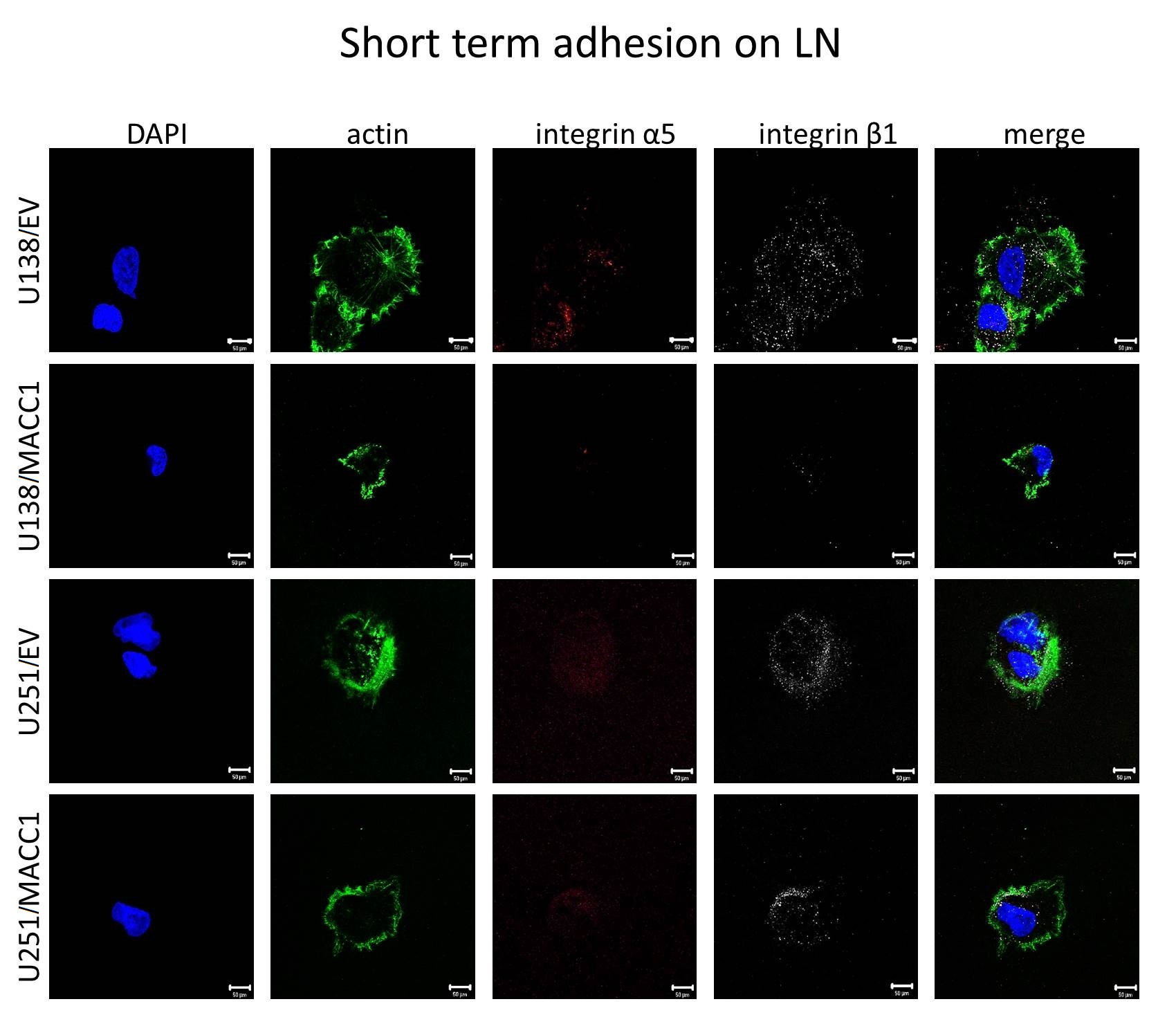

Supplement: Supplementary file 11 — Additional file 10: Figure S6. Integrin α5 and β1 distribution on LN for cells allowed to adhere for 30 min. Integrins were mainly localized near the nucleus or the expanding actin cytoskeleton. No significant MACC1-dependent change in integrin distribution could be observed. n > 9. Scale bar depicts 50 μm. [file 12964_2020_566_MOESM11_ESM.tif]

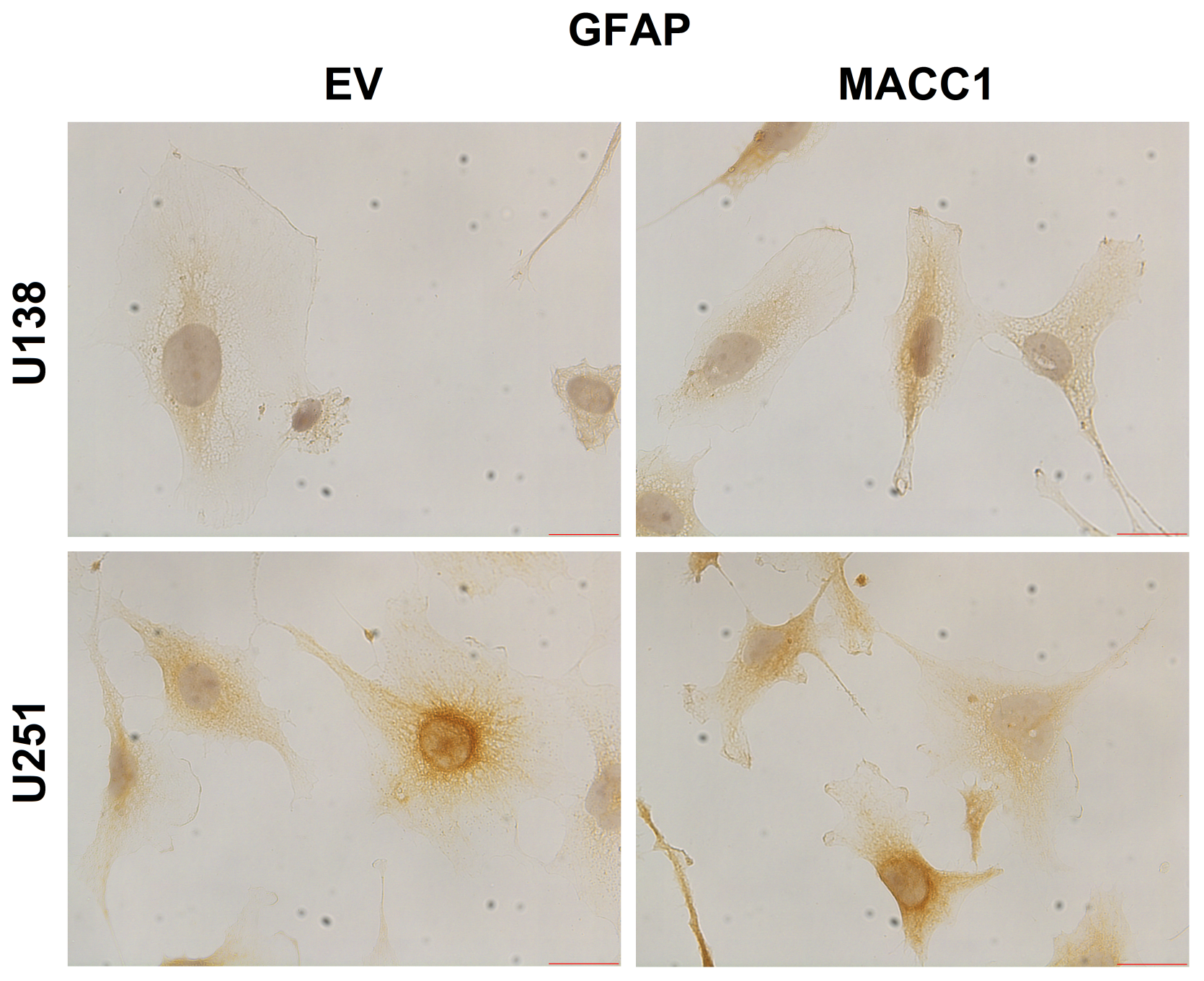

Supplement: Supplementary file 12 — Additional file 11: Figure S7. Staining of U138 and U251 cells for GFAP. MACC1 overexpression was not associated with a visible change in GFAP organization. Scale bar corresponds to 25 μm. [file 12964_2020_566_MOESM12_ESM.tif]

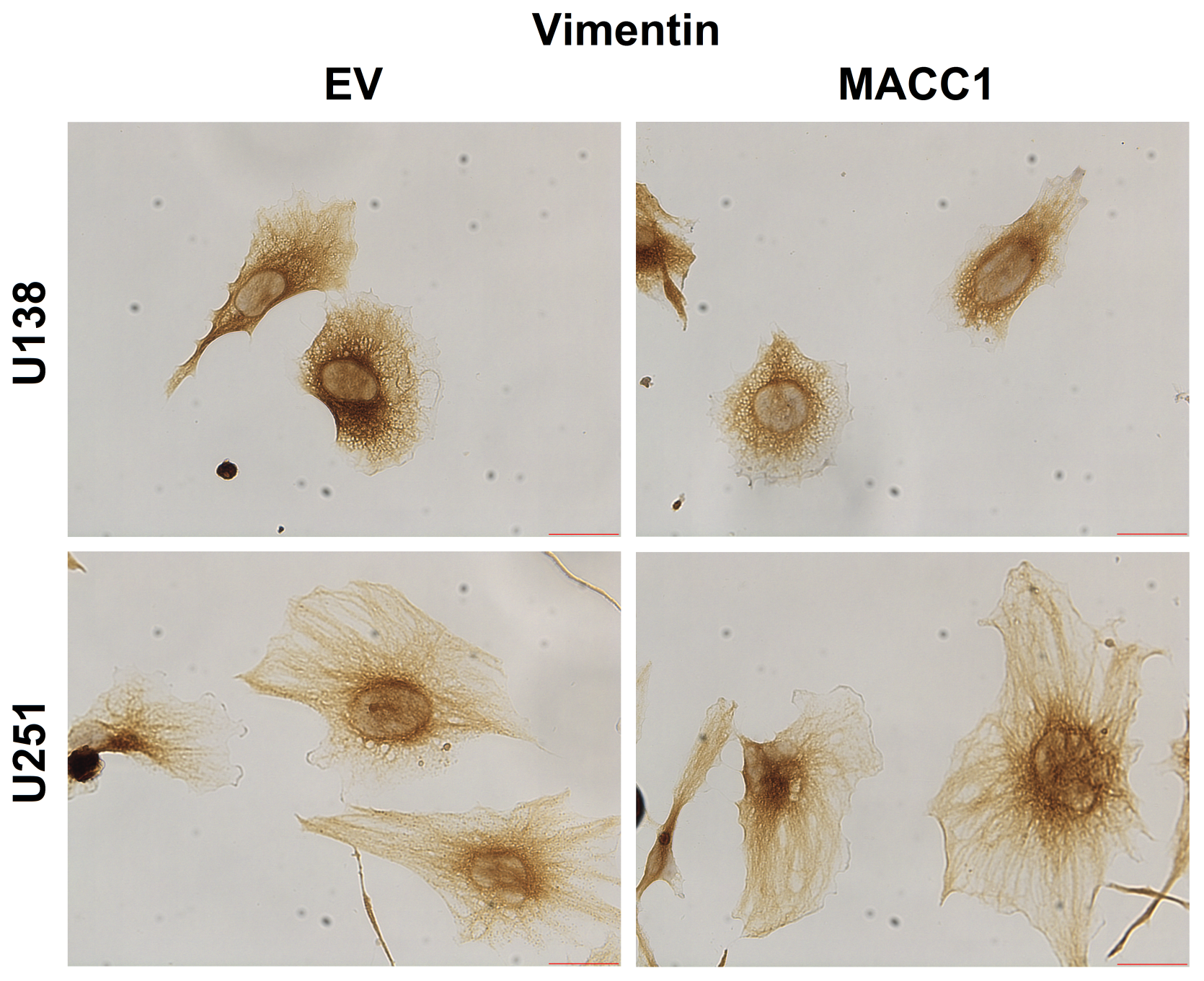

Supplement: Supplementary file 13 — Additional file 12: Figure S8. Staining of U138 and U251 cells for vimentin. MACC1 overexpression was not associated with a visible change in vimentin organization. Scale bar corresponds to 25 μm. [file 12964_2020_566_MOESM13_ESM.tif]

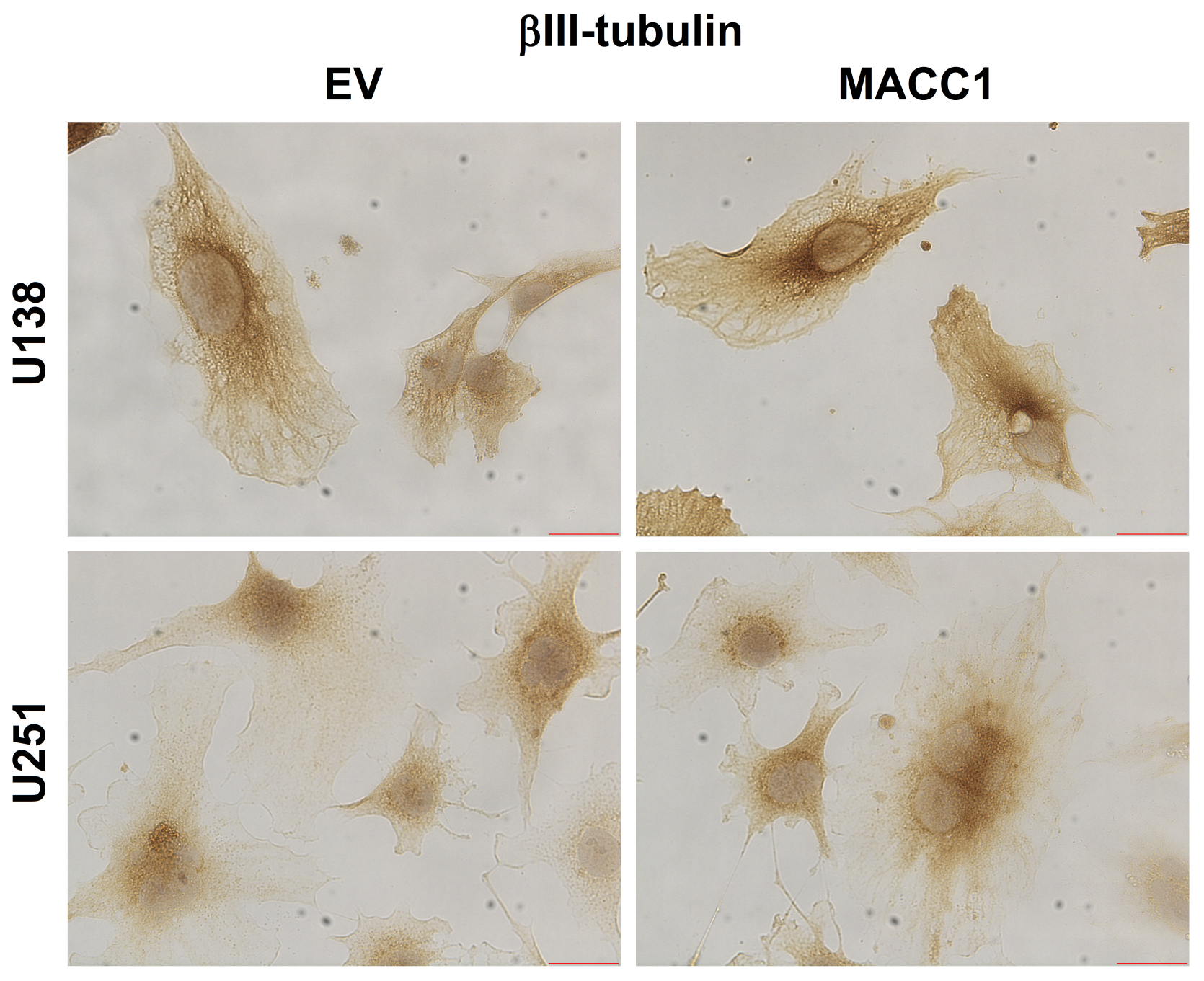

Supplement: Supplementary file 14 — Additional file 13: Figure S9. Staining of U138 and U251 cells for βIII-tubulin. MACC1 overexpression was not associated with a visible change in microtubule organization. Scale bar corresponds to 25 μm. [file 12964_2020_566_MOESM14_ESM.tif]
