# Supplementary material for: MACC1 driven alterations in cellular biomechanics facilitate cell motility in glioblastoma
Source: Cell Commun Signal. 2020 Jun 5;18:85. doi: 10.1186/s12964-020-00566-1 (PMC7275321; doi:10.1186/s12964-020-00566-1)
Supplement: Supplementary file 15 — Additional file 14: Supplemental Results. Estimation of cell-cell adhesion from 3D aggregate formation. [file 12964_2020_566_MOESM15_ESM.docx]

**Supplemental Results:**

**Estimation of cell-cell adhesion from 3D aggregate formation:**

Plugging in all values measured for our experiments and with *ν* = 0.5 the model gives reasonable results in the order of 1.4-2.6 mJ/m², comparable to those obtained by Frasca et al. (1).

The respective adhesion energy ratio for U138/EV to U138/MACC1 is ≈1.12 and for U251/EV to U251/MACC1 ≈1.42, indicating a lower adhesion in *MACC1* overexpressing cells.

The exact values used for the calculation are found in the table below:

| Parameter | U138/EV | U138/MACC1 | U251/EV | U251/MACC1 |
| --- | --- | --- | --- | --- |
| E [Pa] | 1690 | 2160 | 1327 | 1610 |
| d [µm] | 20.9 | 17.5 | 20.2 | 17.5 |
| A_0_ | 1 | 1.445 | 1 | 0.825 |
| A_∞_ | 0.240 | 0.374 | 0.086 | 0.119 |
| I_0_ | 0.405 | 0.430 | 0.591 | 0.630 |
| I_∞_ | 0.097 | 0.083 | 0.086 | 0.117 |

**Bibliography:**

1. Frasca G, Du V, Bacri J-C, Gazeau F, Gay C, Wilhelm C. Magnetically shaped cell aggregates: from granular to contractile materials. Soft Matter. The Royal Society of Chemistry; 2014;10(28):5045.
